# Intraosseous schwannoma of the proximal humerus with pathologic fracture

**DOI:** 10.1186/s40001-021-00541-7

**Published:** 2021-07-09

**Authors:** Jiang Huajun, Qu Wei, Wu Yuxuan, Yang Jingjing

**Affiliations:** 1 Department of Orthopaedics, First Affiliated Hospital of Dalian Medical University, No. 222, Zhongshan Road, Dalian, 116011 China; 2 Department of Neurology, First Affiliated Hospital of Dalian Medical University, No. 222, Zhongshan Road, Dalian, 116011 China

**Keywords:** Intraosseous schwannoma, Proximal humerus, Radiographs, Pathological examination, Immunohistochemistry

## Abstract

**Background:**

Intraosseous schwannomas are extremely rare in the humerus, and less than five cases have been reported previously in the literature. This is the first report of its origin in the proximal humerus with pathologic fracture. We herein present this case to discuss the reason for its rarity and share our experience of management.

**Case presentation:**

A 55-year-old female patient presented with pain in the right shoulder, which was caused by tripping and falling over a board. Radiographs, computed tomography (CT) and magnetic resonance imaging (MRI) showed considerable tumor in proximal humerus, which connected with a fracture. For this suspected tumor, we performed two operations. Pathological examination demonstrated typical picture of a schwannoma, showing whorls and interlacing fascicles of schwannoma spindle cells. Immunohistochemistry, the tumor cells were diffusely positive for S-100 protein, SOX-10 and CD68, while they were completely negative for desmin, DOG-1, AE1/AE3 and P63. The Ki-67 index was about 10%. No mitoses or features of malignancy were identified. The final diagnosis of intraosseous schwannoma was made. The treatment for intraosseous schwannoma with pathologic fracture includes excisional biopsy, curettage, bone allograft, and fracture fixation. The patient recovered well. After the surgery, the patient gradually regained mobility and the pain subsided. There was no recurrence after 6 months of follow-up by X-ray.

**Conclusions:**

Although very rare, intraosseous schwannoma should be taken under consideration in the differential diagnosis of benign-appearing osseous tumor in the proximal humerus with pathologic fracture.

## Background

Schwannoma is a neurogenic tumor that arises from a local proliferation of schwannoma cells in the peripheral, cranial, or visceral nerve. Intraosseous schwannomas account for less than 0.2% of primary bone tumors [1], and the most common site for intraosseous schwannoma is the mandible. To our knowledge, Intraosseous schwannomas are extremely rare in the humerus, and less than five cases have been reported previously in the literature [2–5]. This is the first report of its origin in the proximal humerus with pathologic fracture. We herein present this case for discussing the reason for its rarity and sharing our experience of management.

## Case presentation

A 55-year-old female patient was referred to our hospital with right shoulder pain caused by tripping and falling over a board on the same day. She had pain and restriction of movements of her right shoulder joint. Her history was notable for having antecedent pain in the right shoulder with restriction of overhead movements for about 1 year. She denied previous shoulder injury, or surgery. On presentation, the patient complained about right shoulder pain, swelling and ecchymosis. On palpation, tenderness was noted all around the shoulder. The skin of the shoulder was intact, and there was no evidence of warmth, erythema, or induration. Both neurological and vascular examinations were normal.

**Fig. 1:**
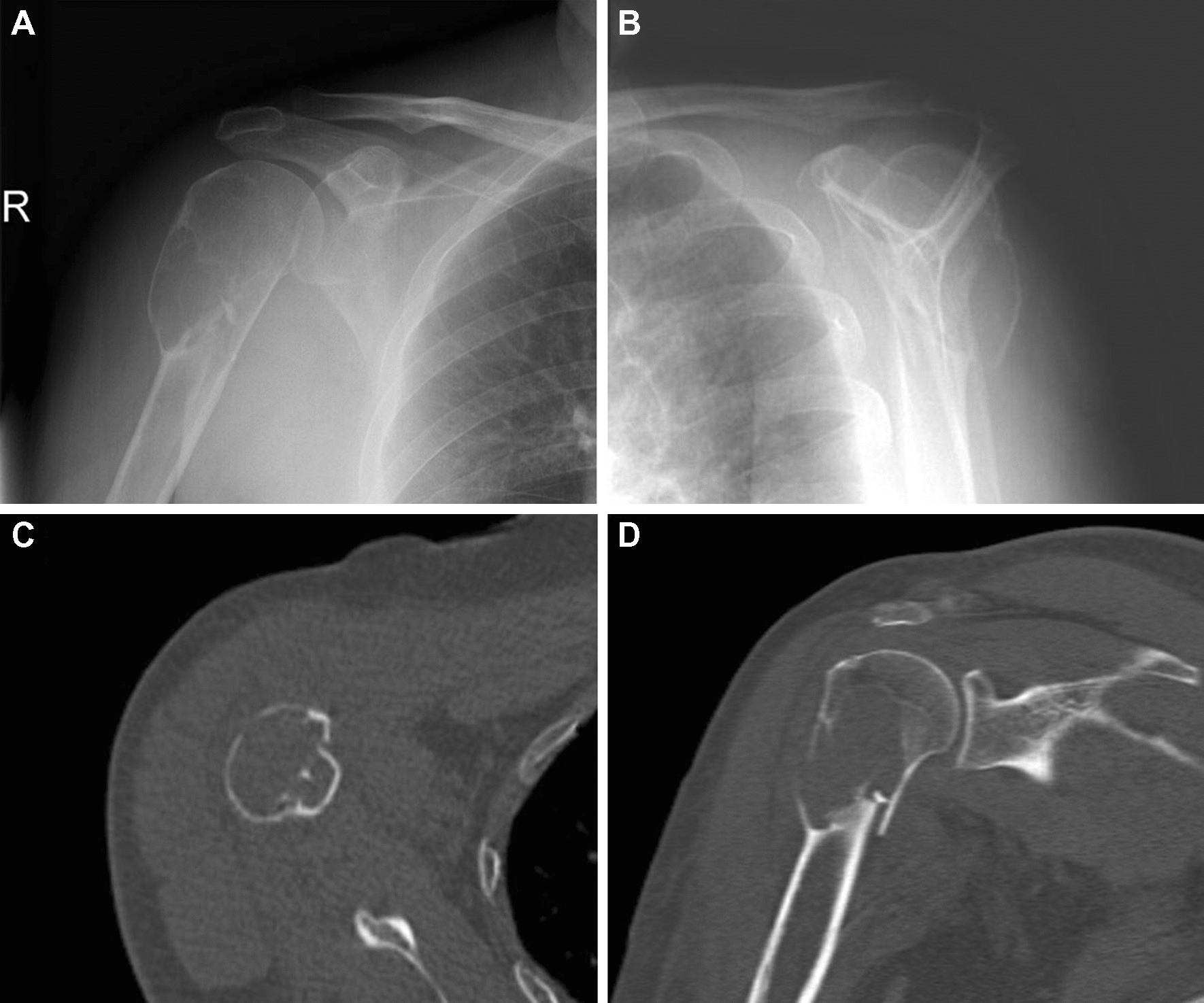
Anteroposterior (**A**) and lateral (**B**) radiographs, shows an expanding, lytic lesion affecting the proximal humerus associated with scalloping of the endosteum. Computed tomography (CT) axial slice bone window (**C**) shows a intramedullary mass elevating the periosteum. Coronal slice bone window (**D**) shows a fracture at the proximal humerus region

Radiographs of the right shoulder showed a large, well-defined osteolytic tumor, which gave an appearance of endosteal scalloping and trabeculated contours on the edge of the bone lesion, involving the proximal humerus (Fig. 1A, B). No significant periosteal reaction was present, and no soft tissue mass or central calcifications were found.

Computed tomography (CT) showed considerable well-defined osteolytic lesion with cortical ballooning and thinning. The density of the tumor was homogeneous and had invaded into the cortex. An intramedullary mass elevating the periosteum was seen at the axial slice bone window and a fracture was seen at the coronal slice bone window (Fig. 1C, D).

**Fig. 2:**
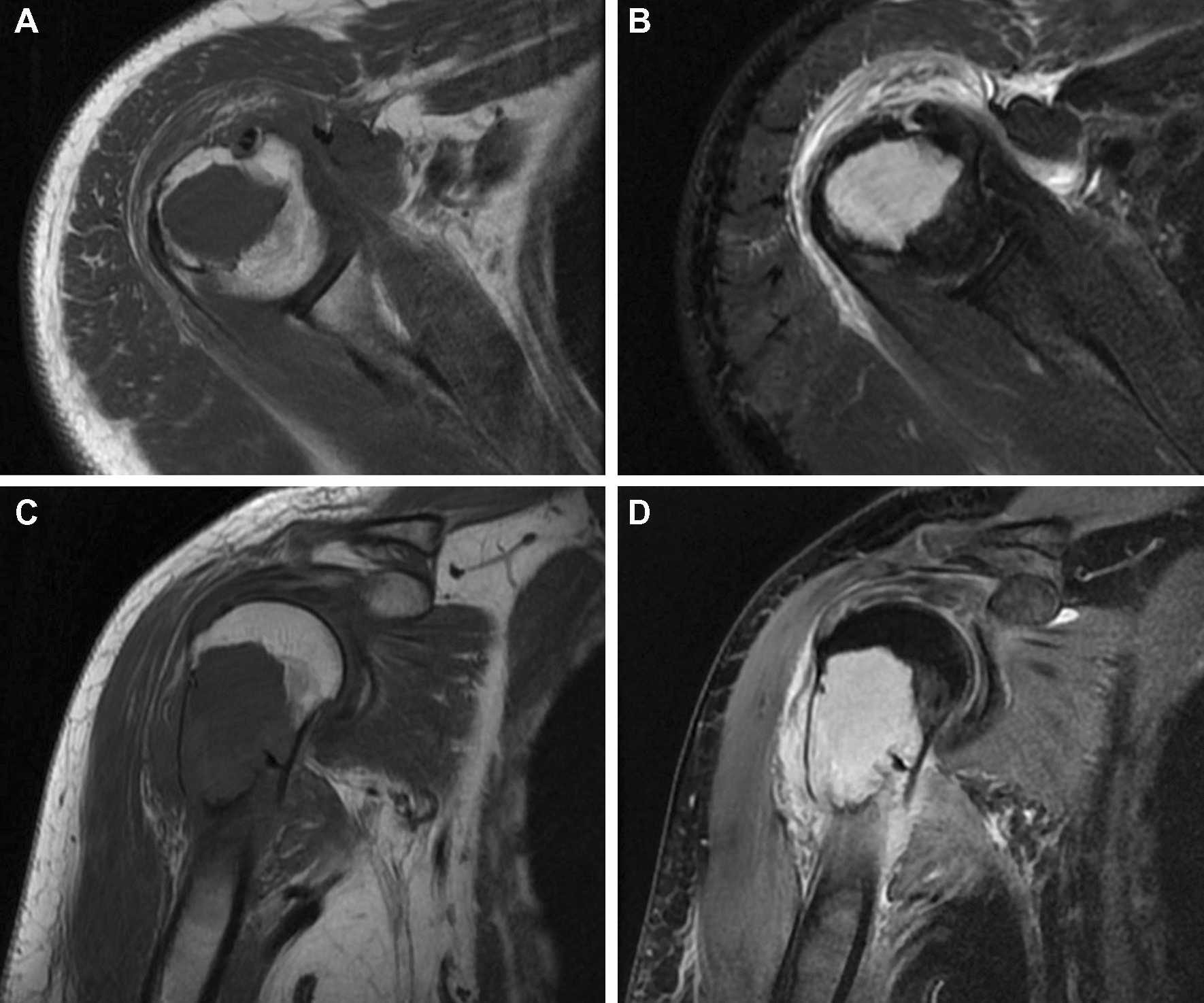
T1-weighted image shows an intramedullary lesion, isointense to muscle, **A** axial image, **C** coronal image. T2-weighted image shows a hyperintense lesion associated with soft tissue edema, **B** axial image, **D** coronal image

Magnetic resonance imaging (MRI) of the right shoulder showed the tumor had invaded into the cortex and the bone lesion with associated soft tissue edema. The lesion was isointense to muscle on T1-weighted images (Fig. 2A, C), and hyperintense on T2-weighted images (Fig. 2B, D). It was noted to involve the anterior, medial, posterior aspect of proximal humerus. The shoulder joint space was uninvolved.

**Fig. 3:**
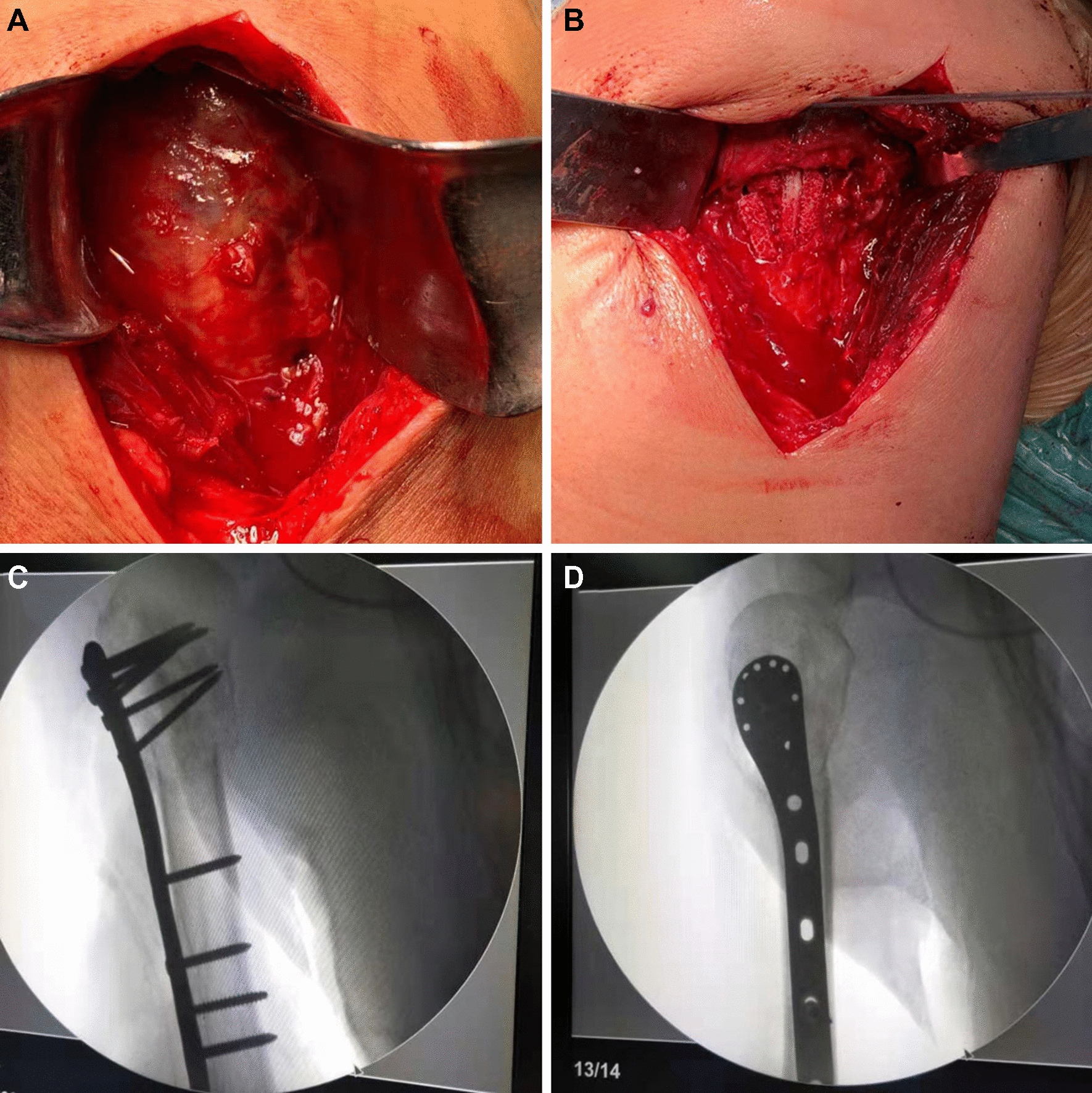
**A **Intraoperative, the tumor was a well-encapsulated soft grayish-yellow colored mass, **B** the tumor was removed and the cavity was filled with allograft bone. **C**, **D** the pathologic fracture was fixated with a proximal humerus locking plate.

Because of uncertainty regarding the histological origin of the tumor, we performed an open biopsy. The operation was performed using a standard deltopectoral approach to access the proximal humerus. Intraoperative the anterolateral cortex of the proximal humerus was elevated (cortical flap). After exposing and removing the upper bony sheath, we found that the tumor was a well-encapsulated soft grayish-yellow colored mass (Fig. 3A). The pathological tissue was removed and sent for histopathological analysis.

**Fig. 4:**
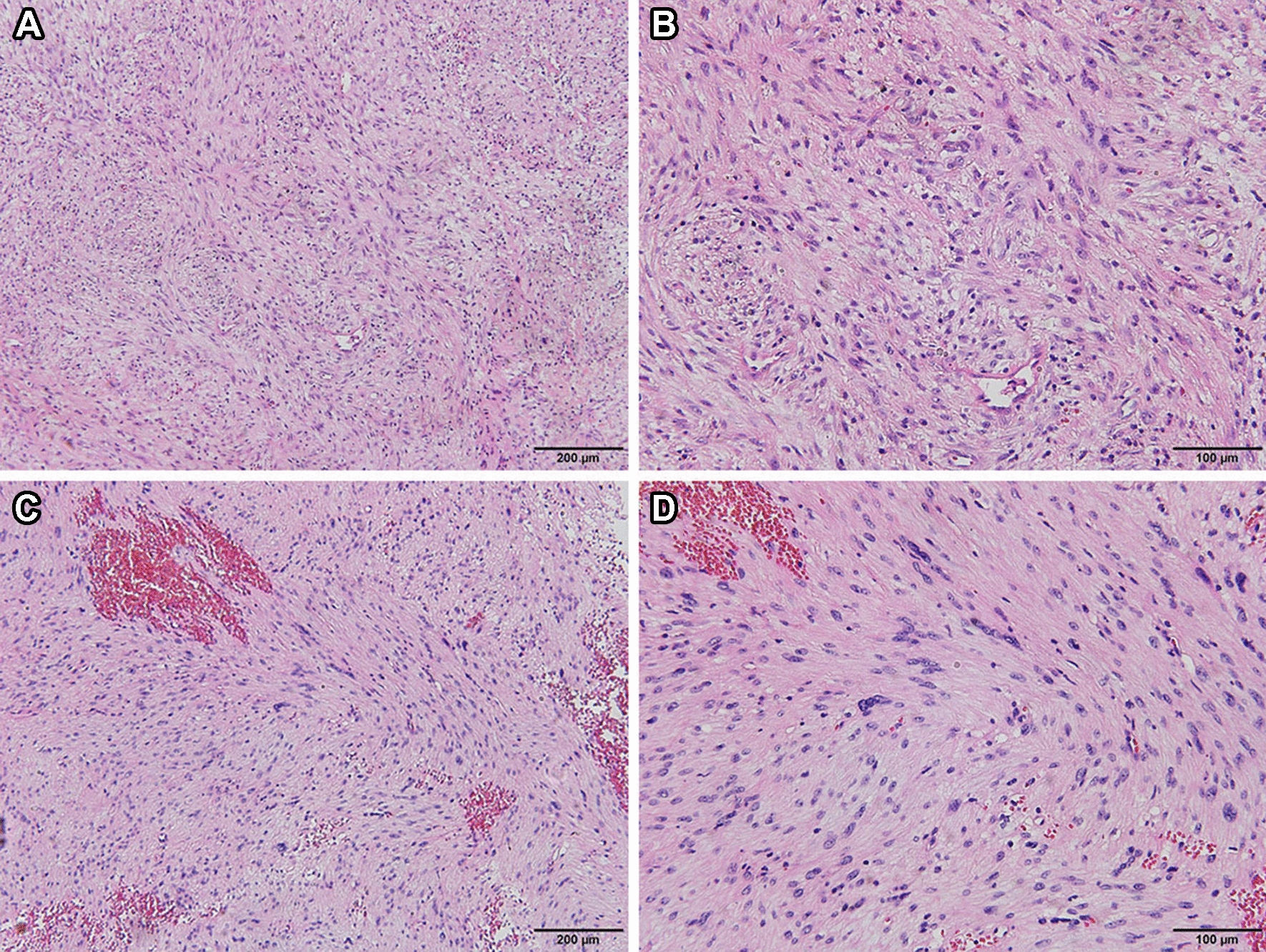
Pathological examination demonstrated typical picture of schwannoma, showing whorls and interlacing fascicles of schwannoma spindle cells (**A**: HE 100; **B**: HE 200). Some specimens showed intratumoral bleeding (**C**: HE 100; **D**: HE 200)

**Fig. 5:**
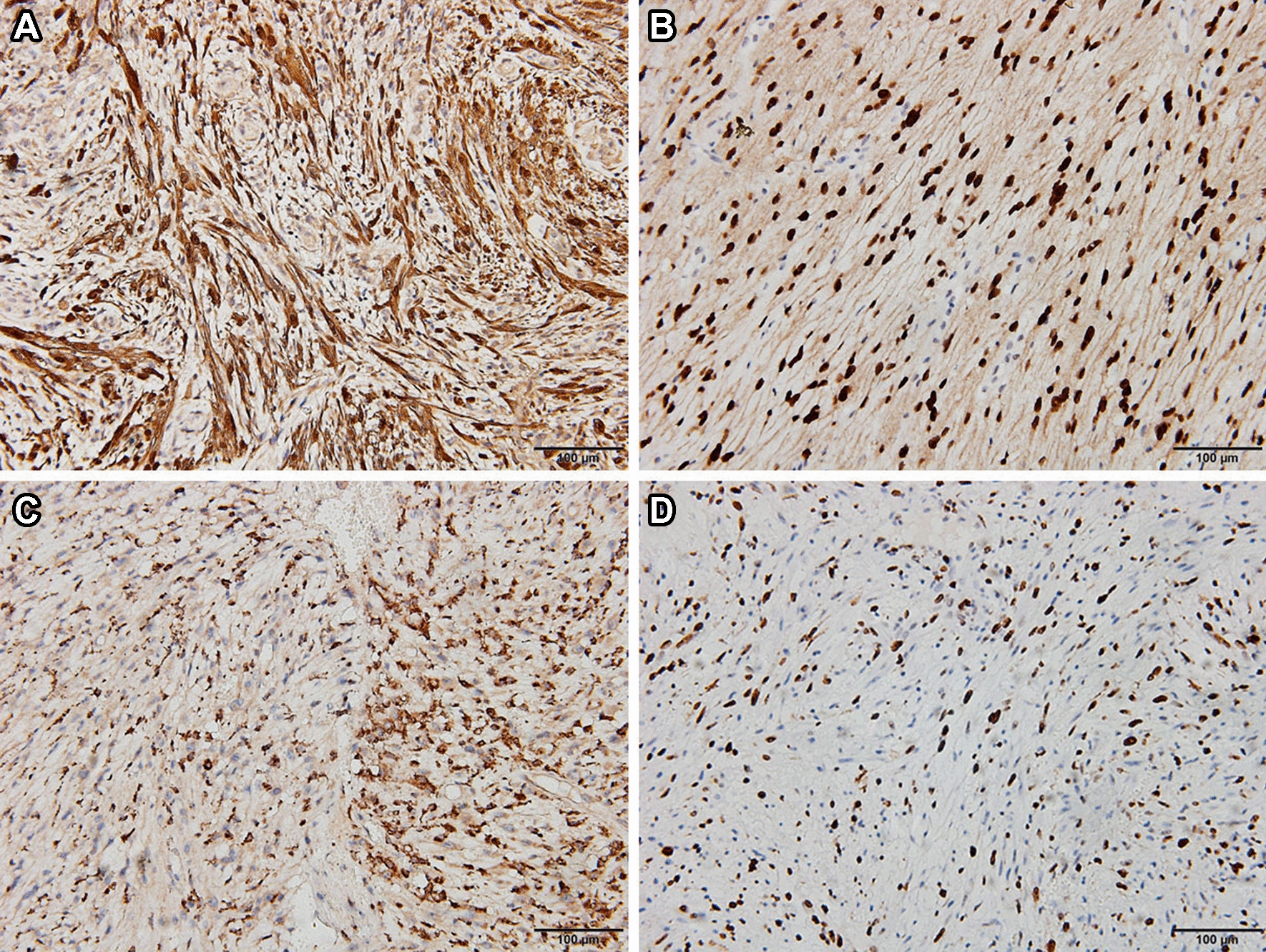
Immunohistochemical analysis demonstrated the presence of **A** S-100, **B** SOX-100, **C** CD68 and **D** Ki-67(+ 10%)

**Fig. 6:**
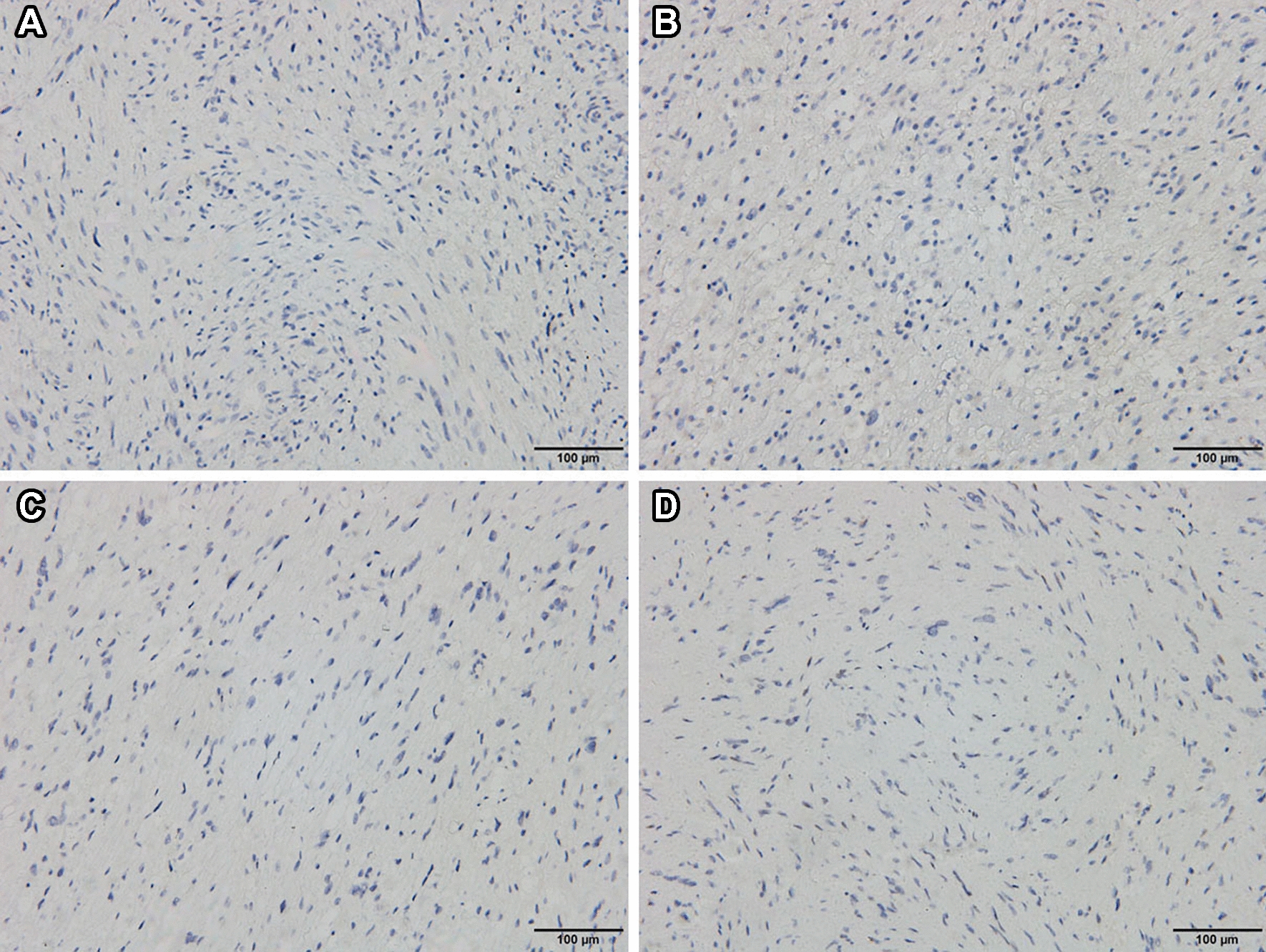
Immunohistochemical analysis demonstrated the absence of **A** Desmin, **B** DOG-1, **C** AE1/AE3, **D** P63

Pathological examination demonstrated typical picture of a schwannoma, showing whorls and interlacing fascicles of schwannoma spindle cells (Fig. 4A, B), and some specimens showed intratumoral bleeding (Fig. 4C, D). Immunohistochemistry for S-100 protein, SOX-10, CD68, desmin, DOG-1 (discovered on gastrointestinal stromal tumors-1), AE1/AE3, P63, Ki-67 was performed. The tumor cells were diffusely positive for S-100 protein, SOX-10 and CD68 (Fig. 5A–C), while they were completely negative for desmin, DOG-1, AE1/AE3 and P63 (Fig. 6A–D). The Ki-67 index was about 10% (Fig. 5D). No mitoses or features of malignancy were identified. Finally, a diagnosis of benign schwannoma with focal of actively proliferated cells was made.

Then the patient was scheduled 10 days after the biopsy for an extended curettage, bone allograft, and fracture fixation, using the same approach. Intraoperative, a complete and meticulous curettage was performed, the cavity was filled with allograft bone (Fig. 3B), and the pathologic fracture was fixated with a proximal humerus locking plate (Fig. 3C, D).

The patient recovered well. After the surgery, physiotherapy was started the day after the operation and a sling was given for comfort. The patient gradually regained mobility and pain subsided. There was no recurrence after 6 months follow-up by X-ray.

## Discussion

Intraosseous schwannomas are rare benign neoplasms of the bone of which fewer than 200 cases have been described in the world literature [6]. In all of these, the majority of intraosseous schwannomas have been located in the mandible. Kito M and his colleagues have suggested that the high frequency in the mandible is not because of the long intraosseous course, but because the mandibular nerve consists of sensory nerves of the trigeminal nerve origin [7]. On the contrary, most intraosseous nerves are non-myelinated and participate in vasomotor functions. This may be the cause of schwannoma rarely arising in the bones of the extremities [8]. Up until now, less than five cases [2–5] have previously been reported in the humerus. In this paper, we present the first case report of an intraosseous schwannoma affecting the proximal humerus with pathologic fracture.

Clinically, intraosseous schwannoma may be present for years before becoming symptomatic, and pain may be present in about 50% of the cases, whereas no symptoms are present in about 25% [9]. They are often discovered as an incidental finding [10]. In our case, the tumor was found after the incidence of a trivial trauma, even though the patient having antecedent pain in the right shoulder with restriction of overhead movements for about 1 year.

Preoperatively, the possibility of intraosseous schwannoma was not considered in our case because of its rarity and nonspecific clinical and radiological findings. The typical radiographic appearance of intraosseous Schwannoma is a well-defined lytic lesion, sclerotic margins, lobulated or trabeculated contours, cortical expansion, and absence of central calcification [7, 11]. Computed tomography facilitates the detection and delineation of these tumors, and MR imaging shows an isointense signal to muscle on T1-weighted images and homogeneously or heterogeneously hyperintense to fat on T2-weighted images [12]. These characteristics were all manifest in our case. However, these findings are nonspecific [13]. The final diagnosis was not made until histologic examination of tissue obtained.

Pathologically, intraparenchymal schwannomas have similar appearances to soft tissue schwannoma and demonstrate two types of cell arrangements, alternating cellular (Antoni A) and myxoid (Antoni B) areas [14]. Immunohistochemical staining can facilitate the diagnosis. In this study, we performed several immunohistochemical stainings, including S-100 protein, SOX-10, CD68, desmin, DOG-1, AE1/AE3, P63 and Ki-67, which are used as ancillary tests for diagnosis of the tumor. Diffuse immune reactivity for S100 protein and SOX-10 is indicative of schwann cell origin. Negative staining for smooth muscle marker desmin [15], gastrointestinal stromal marker DOG-1 [16, 17], cytokeratin (AE1/AE3) [18], myoepithelial marker p63, respectively [19], are used to rule out histological differential diagnoses. In our case, pathological examination demonstrated typical picture of schwannoma, showing whorls and interlacing fascicles of schwannoma spindle cells. The diffuse immune reactivity for S-100 protein is indicative of a Schwann cell origin. The final diagnosis of intraosseous schwannoma was made.

It is remarkable that the tumor in our case showed higher CD68 staining. Generally, the degree of inflammation measured by the expression of CD68 showed a positive significant correlation with tumor size and tumor growth index [20]. This is inflammatory in nature and may contribute to the pathogenesis of schwannoma [21]. Although there is a lack of clear insight regarding the control mechanisms for oncogenesis, in our case, the tumor with higher CD68 staining likely demonstrates that the tumor volume increase is not only based on cell proliferation, but also intratumoral hemorrhage, vascularization, and inflammation, which may be produce rarefaction of the bone and lead to bone fracture after a trivial trauma.

Ki-67 is a monoclonal antibody that provides a means of evaluating the growth fraction of normal and neoplastic human cell populations. A Ki-67 index of less than 3% is expected for a typical schwannoma. schwannomas with an index of greater than 3% are presumed to be actively proliferating and pose a theoretically higher risk for regrowth or recurrence [22]. However, Ki-67 proliferative index is variable. In a study by Imagama et al., Ki-67 index was < 1% in purely intramedullary tumors while it ranged between 18 and 25% in cases where the tumor was both intra- and extramedullary [23]. In our case, the tumor was both intra- and extramedullary. The Ki-67 index was about 10%, which are presumed to be non-actively proliferating and pose a lower risk for recurrence.

Finally, the recommended treatment for intraosseous schwannomas with pathologic fracture includes excisional biopsy, curettage, bone allograft, and fracture fixation. In our case, there was no evidence of recurrence and have a good functional outcome.
